# Immunopathogenesis of MEWDS and the white dot syndromes: anatomical specificity and mechanistic heterogeneity

**DOI:** 10.3389/fmed.2026.1979898

**Published:** 2026-10-27

**Authors:** Xiaohong Wang, Zhaohui Li, Weiwei Kong, Tingting Yin, Zhihua Cui

**Affiliations:** 1 Department of Ophthalmology, The First Hospital of Jilin University, Changchun, China; 2 Department of Ophthalmology, The Fourth Affiliated Clinical Hospital of Changchun University of Traditional Chinese Medicine, Changchun, China

**Keywords:** choriocapillaris, immunopathogenesis, MEWDS, multimodal imaging, MUV task force, photoreceptor, retinal pigment epithelium, white dot syndromes

## Abstract

The white dot syndromes (WDS) are an umbrella term for a heterogeneous group of idiopathic inflammatory chorioretinopathies—including multiple evanescent white dot syndrome (MEWDS), acute posterior multifocal placoid pigment epitheliopathy (APMPPE), birdshot chorioretinopathy, multifocal choroiditis (MFC), and punctate inner choroidopathy (PIC). MEWDS is the subtype in which the debate over the primary anatomical site of disease initiation has been most intense: three competing hypotheses—choriocapillaritis, photoreceptoritis, and retinal pigment epithelium (RPE) dysfunction—remain unresolved. MEWDS has also accumulated substantial evidence for viral triggers, including SARS-CoV-2 infection and COVID-19 vaccination, whereas other WDS subtypes exhibit distinct mechanistic signatures (HLA-A29 in birdshot, choriocapillaris ischemia in APMPPE, Bruch's membrane disruption in MFC/PIC). This narrative review synthesizes the three MEWDS-specific hypotheses against multimodal imaging, genetic, and immunological evidence published through 2026, and contrasts them with disease-specific mechanisms across the WDS spectrum. Indocyanine green angiography (ICGA) findings support primary choriocapillaritis, whereas optical coherence tomography angiography (OCT-A) has yielded paradoxically preserved flow maps; RPE dysfunction remains plausible given fundus autofluorescence patterns and the RPE's immunoregulatory role. The Multimodal Imaging in Uveitis (MUV) Task Force Report 11 (2026) proposes an anatomical reclassification that assigns MEWDS to the photoreceptor/RPE group. Across WDS, the evidence supports mechanistically distinct diseases and motivates disease-specific, mechanism-driven nomenclature.

## 1 Introduction

The white dot syndromes (WDS) represent a clinically heterogeneous group of inflammatory chorioretinal disorders that have challenged ophthalmologists for more than half a century. Gass's original description of acute posterior multifocal placoid pigment epitheliopathy (APMPPE) in 1968 (1) preceded Jampol and colleagues’ characterization of multiple evanescent white dot syndrome (MEWDS) in 1984 (2); Jampol and Wiredu subsequently proposed grouping these and related entities under the WDS umbrella in 1995 (3), and Gass later described acute zonal occult outer retinopathy (AZOOR) as part of the broader “AZOOR complex” (4). This nosological category encompasses conditions united by the ophthalmoscopic presence of small, yellowish-white lesions at the level of the choroid, retinal pigment epithelium (RPE), or outer retina. The prototypic entities include MEWDS, APMPPE, birdshot chorioretinopathy, multifocal choroiditis (MFC), punctate inner choroidopathy (PIC), and AZOOR.

These conditions share several clinical features: they predominantly affect young to middle-aged adults, often present with photopsias, scotomas, and decreased visual acuity, and are frequently preceded by a viral-like prodrome (5). Notably, they arise in patients who are otherwise healthy and without systemic autoimmune disease, which has made them particularly enigmatic from an immunological standpoint. The spontaneous resolution observed in MEWDS and APMPPE contrasts sharply with the chronic, progressive course of birdshot chorioretinopathy and the recurrent, scarring nature of MFC/PIC, suggesting that these conditions are far more diverse than their shared ophthalmoscopic appearance implies (5, 6). The Standardization of Uveitis Nomenclature (SUN) Working Group has established validated classification criteria for several WDS entities using machine learning approaches, further standardizing research definitions (7). MEWDS in particular shows a striking predilection for young females, with approximately 80% of affected patients being women of reproductive age. Additionally, myopia has been increasingly recognized as a potential risk factor for MEWDS, with several studies reporting a higher prevalence of myopia among MEWDS patients compared with the general population.

From an anatomical perspective, the candidate sites of primary involvement span the choroidal vasculature, the retinal pigment epithelium (RPE), and the outer neurosensory retina. Conditions such as APMPPE and serpiginous choroiditis are characterized by primary injury to the choriocapillaris, involving ischemic or inflammatory disruption of this unique vascular bed (8–10). Birdshot chorioretinopathy demonstrates diffuse chorioretinal inflammation strongly associated with HLA-A29 (47–49). Multifocal choroiditis (MFC) and punctate inner choroidopathy (PIC) involve Bruch's membrane disruption with inflammatory communication between the choroid and neurosensory retina (11, 12). MEWDS, the entity at the center of the ongoing debate, has been variously attributed to primary involvement of the choriocapillaris, the RPE, or the photoreceptor ellipsoid zone (EZ), with competing hypotheses supported by different imaging modalities (7–11). Acute zonal occult outer retinopathy (AZOOR) is primarily a photoreceptor degenerative process with distinct clinicopathological features (4, 13, 14). This anatomical heterogeneity is central to understanding why a single pathogenic framework cannot unify these conditions, and it provides the structural basis for the MUV Task Force's recent reclassification effort (15, 16).

While the WDS umbrella has been clinically useful for grouping these conditions, a fundamental question persists: do these entities represent a continuum of a single disease spectrum or entirely distinct pathological entities? This question is not merely academic; it has direct implications for clinical management, therapeutic decision-making, and prognostication. Over the past decade, the introduction of multimodal imaging—particularly OCT angiography (OCT-A), swept-source OCT, fundus autofluorescence (FAF), and enhanced-depth imaging (EDI) ICGA—has fundamentally altered our understanding of these conditions, revealing previously unrecognized anatomical heterogeneity (6, 17). Among the various WDS subtypes, MEWDS has emerged as the entity in which competing claims about the primary site of pathological involvement have been argued most extensively and for the longest period, making it the focal point of the immunopathogenic debate reviewed in this article.

Within MEWDS specifically, three major competing hypotheses have emerged regarding the primary site of pathological involvement. The choriocapillaritis hypothesis, championed primarily by the Herbort group, posits that the primary event is inflammation and non-perfusion of the choriocapillaris, with secondary damage to overlying RPE and photoreceptors (8, 9). The photoreceptoritis hypothesis argues that OCT-demonstrated ellipsoid zone (EZ) disruption represents primary photoreceptor injury, with the RPE and choriocapillaris changes being secondary or epiphenomenal (18, 19). A third, less prominent hypothesis proposes primary RPE dysfunction, with downstream effects on both the choriocapillaris and photoreceptors (2, 20). The anatomical relationships among these three candidate layers are illustrated in Figure 1. Other WDS subtypes have accumulated distinct lines of evidence pointing to different pathogenic mechanisms—for example, the near-universal HLA-A29 association in birdshot chorioretinopathy, primary choriocapillaris ischemia in APMPPE and serpiginous choroiditis, and Bruch's membrane rupture in MFC/PIC—which frame the MEWDS-focused analysis that follows.

**Figure 1:**
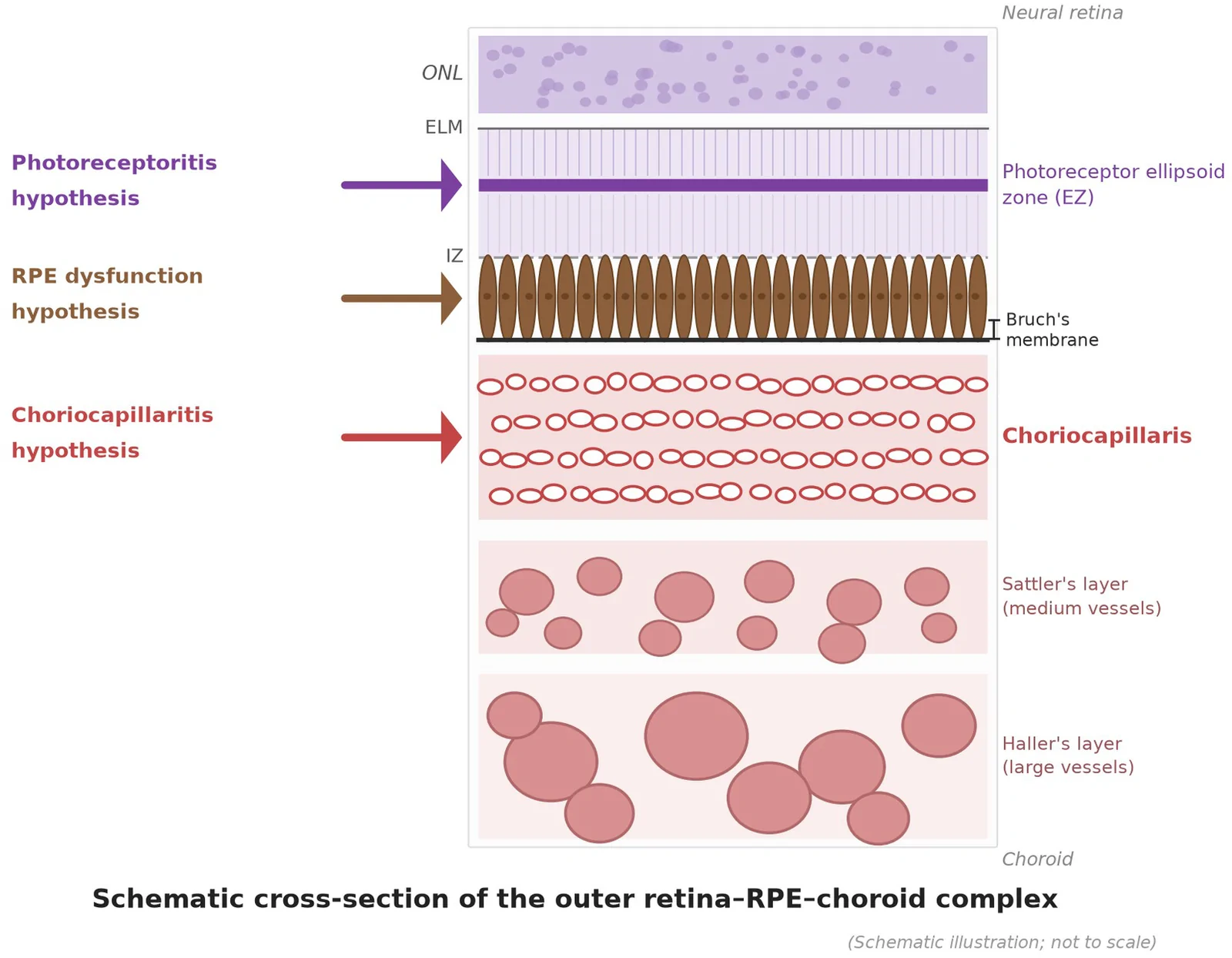
Schematic cross-section of the outer retina–retinal pigment epithelium (RPE)–choroid complex illustrating the three candidate primary sites of injury in MEWDS. The choriocapillaris (bottom, red), RPE monolayer (middle, brown, with apical microvilli ensheathing photoreceptor outer segments), and photoreceptor ellipsoid zone (EZ; top, purple) are separated by Bruch's membrane. Each of the three competing hypotheses—choriocapillaritis, RPE dysfunction, and photoreceptoritis—proposes a different primary anatomical origin. (Schematic illustration; not to scale.).

The most comprehensive recent review of WDS was published by Cicinelli and colleagues in 2023 (6), which provided a broad update on multimodal imaging findings and disease-specific interpretations across the entire WDS spectrum. The present review differs in three deliberate respects. First, rather than surveying all WDS entities equally, we center the unresolved three-hypothesis debate in MEWDS—choriocapillaritis vs. photoreceptoritis vs. RPE dysfunction—and systematically juxtapose the imaging, genetic, and immunological evidence for each position. Second, we integrate the MUV Task Force Report 11 (2026), which postdates the Cicinelli review and introduces an anatomy-based reclassification that assigns MEWDS to a photoreceptor/RPE group while explicitly declining to adjudicate among the sub-retinal layers. Third, we synthesize three converging mechanistic lines—viral triggers, anti-retinal autoantibodies, and HLA susceptibility—that have matured since 2023 and that bear directly on why MEWDS, unlike other WDS entities, remains anatomically contested.

This review critically examines these three competing hypotheses in light of the most recent evidence, explores emerging mechanisms beyond this classical debate—including autoantibodies, viral triggers, and genetic susceptibility—and discusses how the landmark MUV Task Force classification framework is reshaping our approach to these enigmatic conditions. Throughout, we maintain the distinction between MEWDS, where the primary-site debate remains unresolved, and other WDS entities, where disease-specific mechanistic evidence has advanced substantially.

## 2 Search strategy and selection criteria

This is a narrative review. A structured literature search was conducted in PubMed/MEDLINE and supplemented by Scopus and Web of Science from database inception through June 2026. Search terms combined “white dot syndrome,” “multiple evanescent white dot syndrome,” “MEWDS,” “acute posterior multifocal placoid pigment epitheliopathy,” “APMPPE,” “birdshot chorioretinopathy,” “multifocal choroiditis,” “punctate inner choroidopathy,” and “acute zonal occult outer retinopathy” with “immunopathogenesis,” “choriocapillaris,” “photoreceptor,” “retinal pigment epithelium,” “indocyanine green angiography,” “OCT angiography,” “fundus autofluorescence,” “HLA,” “autoantibody,” and “molecular mimicry.” Reference lists of retrieved articles and prior reviews were hand-searched for additional relevant studies.

Articles were eligible if they reported original clinical, imaging, histopathological, genetic, or immunological data relevant to WDS immunopathogenesis, or were authoritative reviews, consensus statements, or classification criteria (including the SUN and MUV Task Force reports). Case reports were included only when they provided mechanistically informative observations not available in larger series. Non-English articles without an English abstract and conference abstracts without subsequent full publication were excluded. No quantitative synthesis was performed; study selection and data extraction were conducted by the authors, with priority given to studies employing multimodal imaging, histopathological confirmation, or immunogenetic methods. Given the narrative design, risk-of-bias assessment was not formally performed, but methodological limitations of individual studies are discussed where they bear on the strength of mechanistic inference. The initial search yielded 1,247 records across all databases. After removal of 312 duplicates, 935 records were screened by title and abstract, of which 748 were excluded as irrelevant. Full-text assessment of 187 articles led to the inclusion of 70 references in the final analysis, comprising original research articles, systematic reviews, consensus statements, and relevant case series.

## 3 The choriocapillaritis hypothesis in MEWDS

***Scope note***. The three-hypothesis debate presented in Sections 3–5 pertains specifically to MEWDS, the WDS subtype in which competing claims about the primary site of injury have been argued most extensively and for the longest period. It is important to note that MEWDS, unlike some other WDS entities, has accumulated substantial evidence for viral triggers (e.g., SARS-CoV-2, HPV vaccination) and demographic predilections, yet the precise anatomical site of disease initiation remains contested. We do not imply that APMPPE/serpiginous choroiditis, birdshot chorioretinopathy, or MFC/PIC share the same primary lesion or the same etiological uncertainties; disease-specific mechanisms, as far as current evidence supports them, are addressed in Sections 6 and 7. Anatomically, MEWDS is the entity for which competing claims about the primary site of injury have been most extensively argued, with the three hypotheses implicating the choriocapillaris, RPE, or photoreceptor EZ respectively. Other WDS entities show more clearly defined primary anatomical involvement (e.g., choriocapillaris ischemia in APMPPE, HLA-A29—associated choroidoretinal inflammation in birdshot, Bruch's membrane breach in MFC/PIC).

### 3.1 Core premise and historical context

The choriocapillaritis hypothesis asserts that the primary pathological event in MEWDS is inflammation-induced non-perfusion of the choriocapillaris, with subsequent ischemic injury to the overlying RPE and photoreceptors (8, 9). This conceptualization emerged from early ICGA observations that choroidal vascular changes preceded and exceeded the extent of clinically visible white dots (21). The concept of primary choroidal vascular involvement in WDS-related entities was also recognized in APMPPE, where Deutman and colleagues, using fluorescein angiography, first proposed that APMPPE represented a choriocapillaritis rather than a primary pigment epitheliopathy, providing early evidence that the choriocapillaris could be the primary site of pathology in APMPPE specifically (10). Whether the APMPPE mechanism extends to MEWDS is precisely what remains contested (see Sections 4, 5, and 7).

The choriocapillaris is a unique vascular bed: it consists of a dense, fenestrated capillary network that supplies the outer retina indirectly through the RPE. ICG (775 Da) is approximately 98% protein-bound, forming a ∼60–80 kDa complex that still extravasates through fenestrated choriocapillaris under physiological conditions (9, 21). This anatomical feature is critical for interpreting ICGA findings: even protein-bound ICG can cross the fenestrated choriocapillary endothelium, and late-phase hypofluorescence cannot be attributed solely to molecular size restriction.

### 3.2 ICGA evidence

Representative multimodal imaging from an acute MEWDS patient is shown in Figure 2. The cornerstone of the choriocapillaritis hypothesis derives from ICGA studies. In MEWDS, ICGA consistently reveals hypofluorescent dark dots (HDDs) that are significantly more numerous and more widely distributed than the white dots visible on clinical examination or color fundus photography (8, 21, 22). Papasavvas and colleagues demonstrated that these HDDs often extend well into the mid-periphery, far beyond the posterior pole lesions visible clinically (5). This discrepancy between the ICGA-identified lesion area and the clinically apparent involvement is perhaps the single most important piece of evidence supporting the choriocapillaritis model: if the disease were primarily confined to the photoreceptor layer, one would expect concordance between clinical and angiographic extent.

**Figure 2:**
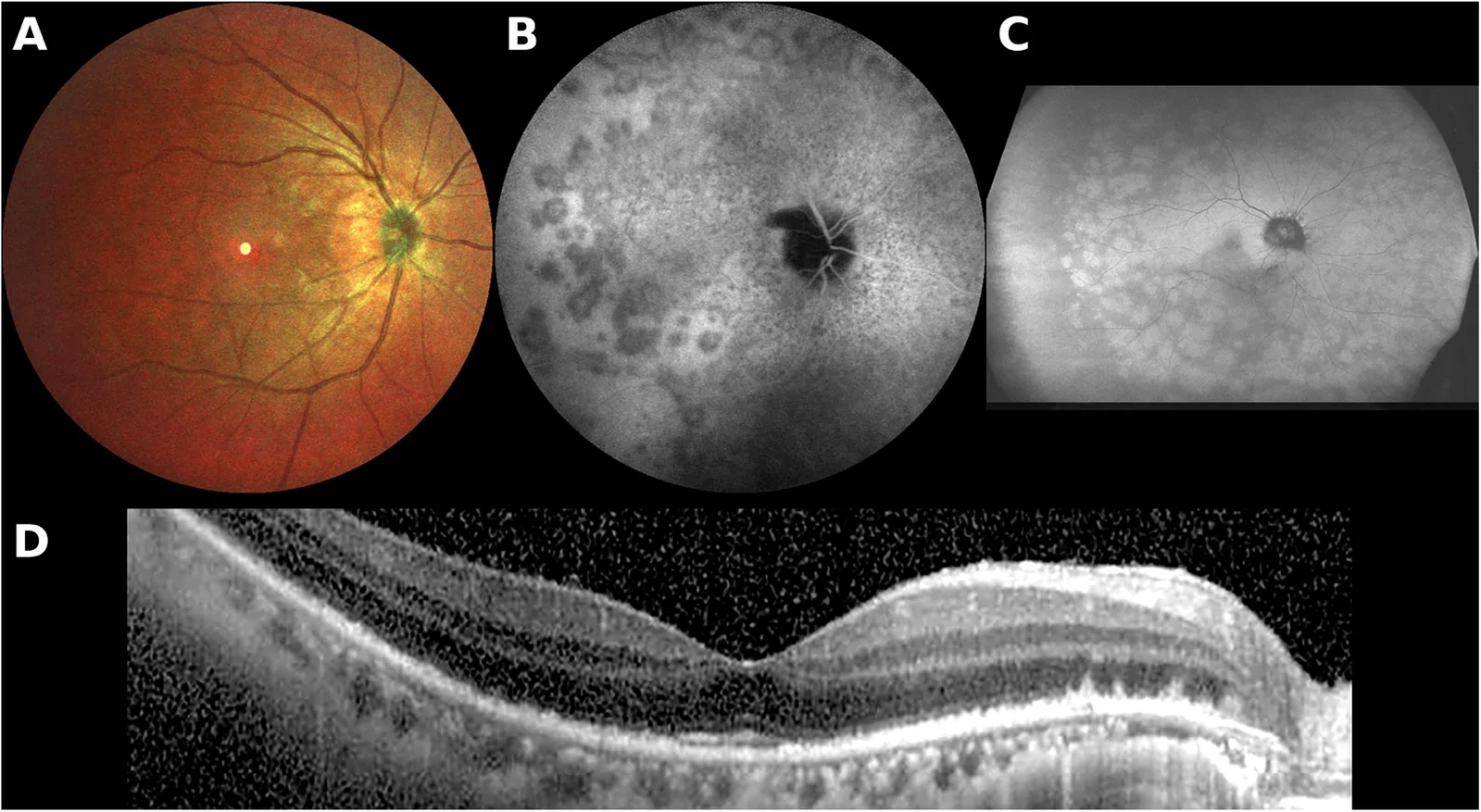
Multimodal imaging findings in acute MEWDS (right eye). **(A)** Multicolor fundus photography showing numerous faint yellowish-white dots at the posterior pole and extending toward the periphery. **(B)** Late-phase indocyanine green angiography (ICGA) showing numerous hypofluorescent dark dots (HDDs) that are more extensive than the clinically visible lesions. **(C)** Fundus autofluorescence (FAF) showing multiple hyperautofluorescent spots at the posterior pole and around the optic disc that correspond to the active lesions. **(D)** Spectral-domain optical coherence tomography (SD-OCT) showing disruption of the ellipsoid zone (EZ) at the level of the photoreceptors, with an intact retinal pigment epithelium band. The discrepancy in lesion extent across modalities—particularly between late-phase ICGA, FAF, and the clinically visible fundus lesions—is central to the ongoing debate regarding the primary site of injury in MEWDS. Panels A and B are from a patient diagnosed and treated at our department (right eye, July 2017); identifying information has been removed and written informed consent was obtained. Panels C and D are from a single patient with acute MEWDS (right eye), so that the FAF abnormalities and the EZ disruption correspond to the same eye at the same disease stage; both panels are reproduced from Miyake et al., Frontiers in Ophthalmology 2025;5:1557294 (CC BY 4.0).

Herbort and colleagues have further argued that the late-phase hypofluorescence observed on ICGA represents true choriocapillaris non-perfusion rather than mere RPE uptake dysfunction (8, 23). This interpretation is grounded in the biophysical properties of ICG: because the dye freely diffuses through the fenestrated choriocapillary endothelium, the late-phase signal depends on the continued presence of dye within the vascular bed. True non-perfusion would manifest as persistent hypofluorescence, whereas mere RPE dysfunction would be expected to show different kinetic patterns (9, 21).

Importantly, the continuity concept has received support beyond the Herbort group. Monferrer Adsuara and colleagues, in an independent observational study, confirmed that ICGA findings in MEWDS demonstrate choroidal vascular changes that exceed the clinically apparent lesions, and further argued that APMPPE represents the more severe end of the same pathophysiological spectrum involving larger choroidal vessels (21). Papadia and Herbort, in their global review of birdshot retinochoroiditis, documented that ICGA reveals more extensive choroidal stromal involvement than is clinically apparent (22). Deutman and colleagues' original angiographic analysis similarly concluded that the weight of fluorescein evidence favors primary choroidal vascular involvement in APMPPE (10), while Papasavvas and colleagues provided a comprehensive multimodal imaging case series of APMPPE that further supports primary choriocapillaris ischemia as the central mechanism (24). The temporal dynamics of lesion evolution in MEWDS—with centrifugal progression from a central epicenter—are also consistent with an inflammatory vascular process spreading within the choriocapillary bed (25).

### 3.3 The OCT-A paradox

The advent of OCT-A introduced an apparent paradox. Several studies using OCT-A have reported seemingly preserved choriocapillaris flow in MEWDS eyes, which would appear to contradict the ICGA-based choriocapillaritis hypothesis (26, 27). However, proponents of the choriocapillaritis model have offered a compelling counter-argument: OCT-A detects flowing blood cells and is critically dependent on flow velocity (26, 28). The choriocapillaris, particularly at its terminal end-arteriole level, may operate at flow velocities below the detection threshold of current OCT-A systems. Consequently, “normal” appearing choriocapillaris on OCT-A may reflect a detection artifact rather than genuine preservation of blood flow (8, 26).

This limitation is not merely theoretical. Herbort has emphasized that OCT-A cannot distinguish between absent flow and flow below the signal threshold, which is a fundamental constraint of the motion-contrast technique (8). The discrepancy between ICGA (which detects dye within the vascular bed through tissue fluorescence) and OCT-A (which detects moving red blood cells) may therefore be methodological rather than pathophysiological (26, 29). However, not all multimodal comparisons have favored the choriocapillaritis interpretation: Labattut and colleagues found that ICGA hypofluorescent areas extended beyond BAF abnormalities and concluded that their findings were more consistent with primary RPE dysfunction than with choriocapillary non-perfusion (20)—a result discussed further in Section 5. Comprehensive reviews of choroidal imaging in uveitis have nonetheless reaffirmed that ICGA remains the gold standard for identifying occult choriocapillary non-perfusion in WDS entities where true ischemia is suspected (30).

### 3.4 Spontaneous recovery

The choriocapillaritis hypothesis also provides an elegant explanation for the characteristic spontaneous recovery observed in MEWDS. If the primary insult is inflammatory occlusion of terminal choriocapillary vessels, then resolution of inflammation would restore perfusion, allowing the metabolically demanding outer retina to recover (5, 9). This is consistent with the observed temporal sequence of EZ disruption followed by gradual recovery, which correlates clinically with the return of visual function (18, 19). The remarkable regenerative capacity of the photoreceptor outer segments, once the underlying ischemic insult is relieved, may explain why MEWDS patients typically regain good visual acuity despite dramatic initial EZ disruption (5, 31).

### 3.5 Limitations

Despite its considerable explanatory power, the choriocapillaritis hypothesis faces several substantive challenges. First, the ICGA late-phase hypofluorescence interpretation, while biophysically plausible, remains inferential—direct histopathological evidence of choriocapillaris non-perfusion in acute MEWDS is still lacking. No biopsy or autopsy study has definitively demonstrated choriocapillary occlusion in acute MEWDS, and given the typically excellent visual prognosis, such tissue may never become available. Second, some OCT-A studies using higher-resolution, swept-source, wider-field protocols have detected flow deficits in at least some MEWDS patients, suggesting that the “normal” choriocapillaris finding may not be universal and that methodological differences between studies may account for some discrepancies (27, 29). Third, the hypothesis struggles to fully account for the presence of anti-retinal autoantibodies and the strong HLA associations seen in certain WDS subtypes, which suggest a more complex, multi-layered immune response (discussed further in Sections 6–7) (32, 33). Fourth, the observation that EZ disruption sometimes precedes clinically detectable choroidal changes on OCT (34) raises questions about the directionality of damage that ICGA alone cannot resolve.

## 4 The photoreceptoritis hypothesis in MEWDS

### 4.1 Core premise

The photoreceptoritis hypothesis posits that the primary site of pathological involvement in MEWDS is the photoreceptor layer itself, specifically the ellipsoid zone (EZ) where photoreceptor inner segments are densely packed with mitochondria (18, 19, 35). This interpretation is driven primarily by OCT findings demonstrating EZ disruption that correlates with areas of functional deficit on microperimetry and multifocal electroretinography (18, 36).

### 4.2 OCT and en-face imaging evidence

High-resolution spectral-domain OCT and, more recently, en-face OCT have revealed that the characteristic white dots of MEWDS correspond to disruptions of the EZ and, in severe cases, the external limiting membrane (18, 19). Cicinelli and colleagues quantitatively demonstrated that photoreceptor reflectivity on OCT accurately aligns with visual function, following a robust nonlinear association across all tested retinal locations, with a centrifugal recovery pattern that mirrors the peripapillary predilection of lesions (19). Wang and colleagues further established the correlation between EZ disruption on en-face imaging and electro-oculographic dysfunction, reinforcing the functional significance of photoreceptor damage (36).

Cahuzac and colleagues described “hyper-early” stage MEWDS in which OCT abnormalities were detectable before the appearance of clinically visible white dots, suggesting that photoreceptor damage may precede the inflammatory signs visible on funduscopy (34). Similarly, Mantovani and colleagues, in a multimodal imaging study of MEWDS foveal granularity, documented disruption of the outer retinal layers on SD-OCT that resolved over time, arguing that these transient photoreceptor-level changes are central to the disease process (35). Labriola and colleagues, using adaptive-optics OCT, further characterized the structure–function relationship, demonstrating that photoreceptor-level disruption corresponds to localized scotomas (37). The MUV Task Force Report 6 (Munk and colleagues) established evidence-based OCT criteria for MEWDS that specifically emphasize the hyperreflective lesions extending from the RPE through the EZ into the outer nuclear layer as a key diagnostic feature (38).

### 4.3 OCT-A and choroidal preservation

Supporters of the photoreceptoritis hypothesis point to OCT-A studies showing preserved choriocapillaris flow in MEWDS as critical evidence against primary choroidal involvement (26, 27). Mebsout-Pallado and colleagues reviewed the literature and concluded that OCT-A findings in MEWDS are heterogeneous, with a substantial proportion of cases demonstrating intact choriocapillaris flow (26). Pradas and colleagues similarly emphasized that OCT-A can successfully differentiate MEWDS from conditions with confirmed choriocapillaris ischemia (27). Swaminathan and colleagues reported swept-source OCT-A findings in MEWDS with inflammatory RPE detachment that showed preserved choroidal vascular flow, further supporting the contention that the choriocapillaris may not be the primary target (39).

### 4.4 Reinterpretation of FA findings

Proponents of the photoreceptoritis hypothesis offer an alternative interpretation of the characteristic wreath-like hyperfluorescence seen on fluorescein angiography (FA) in MEWDS. Rather than reflecting primary choroidal vascular leakage, this pattern may represent secondary changes at the level of the RPE caused by photoreceptor damage (2, 40). The original description by Jampol and colleagues documented visual field disturbances and foveal granularity extending far beyond the clinically visible white dots, suggesting a diffuse outer retinal process—a view conceptually aligned with the photoreceptoritis interpretation (2). Enríquez-Fuentes and colleagues further raised the possibility that some MEWDS cases may actually represent secondary presentations of acute retinal pigment epitheliitis, challenging the notion that all clinically diagnosed MEWDS cases share the same pathogenesis (40).

### 4.5 Limitations of the photoreceptoritis hypothesis

The photoreceptoritis hypothesis, despite its intuitive appeal, faces several important limitations that must be acknowledged.

First, a fundamental limitation is the absence of direct histopathological evidence. Unlike conditions such as acute zonal occult outer retinopathy (AZOOR)—where photoreceptor loss has been documented histologically and multimodal imaging analyses have demonstrated that AZOOR follows a clinicopathological mechanism distinct from the choriocapillaritis entities (13, 14)—no biopsy or autopsy study of acute MEWDS has confirmed primary photoreceptor inflammation. The entire photoreceptoritis argument rests on imaging inference rather than tissue-level proof.

Second, EZ disruption—the central imaging finding cited in support of photoreceptoritis—is not specific to primary photoreceptor injury. EZ disruption can result from multiple mechanisms, including ischemic injury secondary to choriocapillaris non-perfusion, RPE dysfunction affecting photoreceptor outer segment renewal, or inflammatory cytokine-mediated damage. In retinal arterial occlusive diseases, for example, EZ disruption is a well-recognized consequence of inner retinal ischemia transmitted to the outer retina (9). Therefore, the presence of EZ disruption does not, by itself, establish the photoreceptor as the primary target.

Third, some patients demonstrate recovery of EZ integrity on OCT but incomplete recovery of visual function, suggesting that EZ restoration does not necessarily equate to complete photoreceptor functional recovery. This dissociation between structural and functional outcomes challenges the notion that photoreceptor damage is the sole determinant of clinical outcome.

Fourth, the hypothesis does not adequately explain the characteristic ICGA findings. If the primary pathology were confined to the photoreceptors, one would not expect the extensive choroidal hypofluorescence that extends far beyond the clinically visible lesions on ICGA. The photoreceptoritis camp has not provided a convincing alternative explanation for these ICGA observations beyond dismissing them as secondary or epiphenomenal.

Fifth, the concept of outer retinal tubulation (ORT) as a marker of photoreceptor remodeling has been studied extensively by Freund and colleagues in age-related macular degeneration, where ORT represents a neurodegenerative response to photoreceptor injury rather than primary inflammation (41). The application of this concept to WDS requires careful consideration: the presence of ORT-like structures in choriocapillaritis entities such as serpiginous choroiditis, as described by Mateo-Montoya and colleagues (42), may represent a secondary remodeling response to damage initiated at any level—choroidal, RPE, or photoreceptor—rather than evidence of primary photoreceptoritis.

Despite these limitations, the photoreceptoritis hypothesis has attracted support from the broader OCT-focused retinal imaging community, which has increasingly recognized the functional significance of outer retinal changes across many disease entities. The hypothesis correctly emphasizes that photoreceptor damage is a critical determinant of visual outcomes, even if the question of whether this damage is primary or secondary remains unresolved.

## 5 The RPE dysfunction hypothesis in MEWDS

### 5.1 Core premise

The RPE dysfunction hypothesis proposes that the primary pathological event in MEWDS lies within the RPE, with downstream consequences for both the choriocapillaris and photoreceptors (2, 20). This hypothesis draws on observations that RPE changes on imaging can precede or exceed photoreceptor abnormalities, and that FAF patterns suggest primary RPE metabolic dysfunction (2, 20, 43). The direct head-to-head comparison by Labattut and colleagues—finding that ICGA hypofluorescent areas were more extensive than BAF abnormalities in MEWDS and interpreting this pattern as favoring primary RPE involvement rather than true choriocapillary non-perfusion—represents one of the strongest imaging-based arguments for this hypothesis (20).

### 5.2 Fundus autofluorescence evidence

FAF imaging detects lipofuscin within RPE cells as a marker of RPE metabolic activity. In MEWDS, FAF consistently demonstrates hyperautofluorescent spots corresponding to the clinically visible white dots, with areas of hyperautofluorescence that often extend beyond the funduscopically apparent lesions (43, 44). Parmann and colleagues discussed the distinction between primary and secondary elevations in FAF, noting that certain FAF patterns in WDS suggest intrinsic RPE metabolic activation rather than secondary changes driven by photoreceptor or choroidal pathology (43). Wong and colleagues further demonstrated that the combination of OCT and FAF enhances the visualization of WDS lesions and suggests a primary role for RPE dysfunction (44).

The FAF evidence for RPE involvement deserves deeper consideration. Hyperautofluorescence in MEWDS reflects increased lipofuscin accumulation or altered RPE metabolism, both of which imply intrinsic RPE activation. Crucially, FAF abnormalities can be detected in areas where the EZ appears intact on OCT, suggesting that RPE metabolic dysfunction may precede detectable photoreceptor damage in some cases. This temporal sequence—RPE activation preceding photoreceptor disruption—would be consistent with a primary RPE origin. Furthermore, the resolution phase of MEWDS is characterized by a return to normal or near-normal FAF patterns, paralleling the restoration of RPE homeostasis, which is temporally correlated with the recovery of visual function.

### 5.3 ICGA reinterpretation

A key argument of the RPE dysfunction hypothesis is that ICGA late-phase hypofluorescence may reflect impaired ICG uptake by dysfunctional RPE cells rather than true choriocapillaris non-perfusion (2, 20). This interpretation challenges the fundamental premise of the choriocapillaritis model: if the RPE is unable to take up and process ICG normally, the resulting hypofluorescence would mimic the pattern expected from vascular non-perfusion (43). The ICG molecule, after extravasating through the fenestrated choriocapillary endothelium, must be taken up by RPE cells for late-phase visualization; impaired RPE uptake would therefore produce hypofluorescent areas indistinguishable from true non-perfusion on standard ICGA.

### 5.4 RPE as an immunoregulatory cell

An underappreciated line of evidence supporting the RPE dysfunction hypothesis derives from the RPE's established role in maintaining ocular immune privilege. The RPE is not merely a passive barrier or metabolic support cell; it is an active immunoregulatory cell that expresses multiple immunosuppressive molecules. Streilein and colleagues demonstrated that the ocular microenvironment is maintained in part by immunosuppressive factors produced by resident ocular cells, including the RPE (45). The RPE constitutively expresses Fas ligand (FasL), which induces apoptosis of infiltrating Fas-expressing T cells, thereby maintaining immune privilege at the outer retinal interface. The RPE also secretes transforming growth factor-beta 2 (TGF-β2), alpha-melanocyte stimulating hormone (α-MSH), and other neuropeptides that suppress local immune activation and promote regulatory T cell (Treg) differentiation.

When the RPE is dysfunctional or damaged, these immunoregulatory mechanisms may fail, leading to breakdown of immune privilege and permitting an inflammatory cascade in the subretinal space. This framework provides a conceptual bridge between primary RPE dysfunction and the secondary inflammatory changes observed in both the choriocapillaris and photoreceptor layers. In this model, the RPE dysfunction is the initiating event; the choriocapillaris and photoreceptor changes are secondary consequences of immune privilege breakdown and the resulting inflammatory milieu.

### 5.5 The RPE–Bruch's membrane–choriocapillaris complex

An emerging concept that may lend further support to the RPE dysfunction hypothesis is the recognition of the RPE–Bruch's membrane–choriocapillaris complex as a functional unit. In this framework, the RPE, Bruch's membrane, and the choriocapillaris are not independent anatomical compartments but rather an integrated triad in which dysfunction at any level has cascading effects on the others. From this perspective, asking whether the primary defect lies in the RPE or the choriocapillaris may be a false dichotomy: the primary pathology may reside in the unit as a whole, with the relative contribution of each component varying across WDS subtypes or even within individual patients at different disease stages.

Mantovani and colleagues, in their multimodal imaging study of FAF changes in choriocapillaritis, demonstrated that the relationship between RPE dysfunction and choroidal perfusion is bidirectional and cannot be simply unidirectional (28). This observation is consistent with the functional unit concept, in which RPE and choroidal changes are interdependent rather than causally ordered.

### 5.6 Counterarguments and limitations

The RPE dysfunction hypothesis has been substantially challenged by biophysical arguments. The ICG–protein complex (∼60–80 kDa) freely extravasates through the fenestrated choriocapillary endothelium; therefore, late-phase hypofluorescence is more parsimoniously explained by the absence of dye within the vascular bed (i.e., non-perfusion) rather than by impaired trans-epithelial transport (9, 21). Furthermore, Cicinelli and colleagues noted that PIC-like reactions can occur in unrelated retinal diseases, suggesting that some of the imaging findings attributed to primary RPE dysfunction may reflect secondary or reactive changes (11). Belletti and colleagues reviewed near-infrared autofluorescence in non-infectious uveitis and concluded that while RPE changes are prominent in WDS, they likely represent secondary or co-primary rather than sole primary events (46).

The hypothesis also remains underdeveloped in the literature relative to the other two. No large-scale study has specifically tested the RPE dysfunction hypothesis using a multimodal imaging approach designed to distinguish primary RPE from primary choroidal disease. The immunoregulatory role of the RPE, while well-established experimentally (45), has not been specifically invoked in mechanistic studies of WDS pathogenesis. Future research addressing these gaps could substantially strengthen or refute this hypothesis.

### 5.7 Transition: from MEWDS-specific debate to WDS-wide mechanistic diversity

The preceding sections have presented the three competing hypotheses—choriocapillaritis, photoreceptoritis, and RPE dysfunction—that have defined the MEWDS debate for over two decades. The core paradox is that, despite accumulating sophisticated multimodal imaging data, the field cannot yet definitively assign the primary site of injury in MEWDS to one of the three candidate layers. MEWDS has, however, accumulated substantial evidence for specific etiological triggers—viral prodromes in approximately 50% of cases, documented SARS-CoV-2 and COVID-19 vaccination associations, and a striking female preponderance—even as the anatomical origin remains contested.

This situation stands in sharp contrast to other WDS subtypes, where disease-specific mechanistic evidence has advanced considerably. Birdshot chorioretinopathy exhibits one of the strongest gene–disease associations in all of ophthalmology (HLA-A29 in >95% of cases), with a well-characterized IL-17–mediated immune response and diffuse choroidal stromal infiltration originating in the deep choroid (47–49). APMPPE and serpiginous choroiditis show convincing evidence of primary choriocapillaris ischemia, with ICGA-demonstrated choroidal vascular occlusion and, in the case of APMPPE, documented coxsackievirus association (10, 24, 50). MFC and PIC are characterized by breaches in Bruch's membrane with inflammatory communication between the choroid and retina, a mechanistically coherent model that supersedes older unitary concepts (51). In each of these entities, the pathogenic mechanism, while not fully elucidated, has converged toward a more specific anatomical and etiological understanding.

This comparison underscores a critical point: the WDS umbrella encompasses conditions with fundamentally different pathogenic architectures. MEWDS, with its unresolved three-hypothesis debate, represents the exception rather than the rule within this group. The mechanistic diversity across WDS subtypes argues strongly against treating WDS as a single disease entity and motivates the disease-specific, anatomically grounded classification framework examined in subsequent sections.

## 6 Emerging mechanisms beyond the classical debate

### 6.1 Autoantibodies and loss of immune privilege

The classical debate between choriocapillaris, photoreceptor, and RPE as the primary site of disease has recently been complemented—and in some ways superseded—by investigations into the immunological mechanisms driving the inflammatory response. A growing body of evidence implicates anti-retinal autoantibodies in the pathogenesis of WDS and related conditions.

Hashimoto and colleagues compared AZOOR patients with and without anti-retinal antibodies, finding that autoantibody-positive patients exhibited more severe functional impairment and more extensive photoreceptor damage, suggesting a pathogenic role for these antibodies (32). Khanna and colleagues described the multimodal testing characteristics of non-paraneoplastic autoimmune retinopathy (npAIR), demonstrating significant overlap between npAIR and WDS presentations (52). Heckenlively and colleagues provided an earlier comprehensive review of autoimmune retinopathy that established the conceptual framework linking autoantibodies to retinal degeneration (53). Khan and colleagues reported the remarkable co-occurrence of AZOOR and secondary non-paraneoplastic autoimmune retinopathy, suggesting that the boundary between WDS and established autoimmune retinopathies may be porous (54).

The mechanistic basis for autoantibody production in WDS likely involves disruption of the RPE–Bruch's membrane–choriocapillaris complex, which normally serves as an immune-privileged barrier. Inflammatory damage to this barrier could expose normally sequestered retinal antigens to the systemic immune system, triggering an autoimmune cascade—a concept supported by the detection of anti-retinal antibodies in WDS patients (32, 52, 54) and by Streilein's foundational work demonstrating that breakdown of ocular immune privilege permits autoimmune attack on retinal antigens (45).

### 6.2 Viral triggers and molecular mimicry

A striking clinical observation in WDS is the frequent occurrence of a viral-like prodrome preceding ocular symptoms, reported in approximately 50% of MEWDS cases (5, 6). This observation has prompted intensive investigation into the role of viral triggers and the concept of molecular mimicry, wherein viral antigens share epitopes with retinal or choroidal antigens, triggering a cross-reactive immune response.

The COVID-19 pandemic has provided an unprecedented natural experiment. Multiple case reports and systematic analyses have documented MEWDS onset following SARS-CoV-2 infection (55, 56) and, notably, following COVID-19 vaccination (57, 58). Zeng and colleagues conducted a systematic analysis of COVID-19 vaccine–associated MEWDS cases and identified a consistent temporal relationship between vaccination and MEWDS onset, with most cases occurring within one to two weeks of immunization (57). Ramirez Marquez and colleagues documented contralateral recurrences following booster vaccination, providing evidence of re-challenge and reinforcing the causal link (58).

Beyond COVID-19, Providência and colleagues reported serpiginous choroiditis onset after SARS-CoV-2 infection, extending the potential trigger spectrum (56). Carey and colleagues described atypical unilateral multifocal choroiditis and retinal vasculitis following West Nile virus infection (59). Kashyap and colleagues further synthesized the COVID-19 perspective on MEWDS, highlighting shared immunological pathways (60). Li and colleagues documented coxsackievirus exposure in APMPPE with long-term multimodal imaging correlation (50). Ogino and colleagues reported MEWDS following human papillomavirus vaccination, suggesting that immune activation rather than direct viral invasion may be the relevant mechanism (61).

These observations collectively support the molecular mimicry hypothesis: viral antigens (or vaccine-induced immune responses) may trigger cross-reactivity between antiviral immunity and self-antigens at the outer retina/choriocapillaris interface. The specificity of the resulting disease phenotype may be determined by the particular epitope–antibody interactions and the underlying genetic susceptibility of the host (57, 60, 61).

It is important to note that the strength of viral trigger evidence varies considerably across WDS subtypes. In MEWDS, the evidence for viral triggers is the most robust and well-documented: multiple case reports and systematic analyses have established temporal associations between MEWDS onset and both SARS-CoV-2 infection and COVID-19 vaccination (55–58), while HPV vaccination has also been implicated (61). In APMPPE, Li and colleagues documented coxsackievirus exposure with long-term multimodal imaging correlation, providing evidence that viral triggers may also operate in this entity, albeit with less extensive documentation than in MEWDS (50). By contrast, in birdshot chorioretinopathy, the immunogenetic architecture is dominated by the HLA-A29 association, with the ERAP1–HLA-A29 gene–gene interaction providing a mechanistic framework in which antigen processing and presentation, rather than viral molecular mimicry, play the central pathogenic role (47, 48, 62). While viral triggers cannot be entirely excluded in birdshot, the overwhelming genetic predisposition suggests that endogenous antigen presentation, rather than exogenous immune activation, is the dominant mechanism. This differential contribution of viral triggers across WDS subtypes further illustrates the mechanistic heterogeneity that undermines the unity of the WDS umbrella concept.

### 6.3 Toward genetic susceptibility

The observation that only a minority of individuals exposed to the same viral triggers develop WDS strongly suggests that genetic factors modulate susceptibility. This is further underscored by the demographic patterns within WDS subtypes: MEWDS shows a striking female preponderance (∼80% female), while birdshot chorioretinopathy is almost universally associated with HLA-A29 (>95%), providing one of the strongest gene–disease associations in all of ophthalmology.

The immunogenetics of birdshot chorioretinopathy have been intensively studied. Nath and colleagues, using single-cell RNA sequencing, identified a pathogenic CD8(bright) CD244(bright) natural killer cell subset that reflects disease activity in HLA-A29-positive patients (47). Shah and colleagues provided a comprehensive review of the clinical features and immunogenetic basis of birdshot (63). Kuiper and colleagues further elaborated the immunopathogenesis, emphasizing the IL-17–mediated immune response (48). Brézin and colleagues established the foundational HLA-A29 association (49). Loeliger and colleagues demonstrated that HLA-A29 homozygosity increases both susceptibility and severity (64). Gelfman and colleagues identified the critical ERAP1–HLA-A29 gene–gene interaction, showing that two copies of HLA-Aw19 alleles further increase risk in HLA-A29 carriers, providing a model for how antigen-processing genes modulate HLA-associated disease risk (62). Although HLA-A29 positivity is considered a near-pathognomonic feature of BSCR, rare HLA-A29-negative cases have been reported—such as the vitiligo-associated birdshot-like chorioretinopathy described by Krzemińska and colleagues (65)—and the disease has also been documented in an HLA-A29-positive Asian patient, expanding the demographic spectrum beyond the predominantly Caucasian populations initially described (66). Comprehensive reviews by Bousquet and colleagues and others summarize the full clinical and immunogenetic landscape of BSCR (67).

A proposed “three-hit” working hypothesis integrates these observations: a genetic predisposition (HLA haplotype plus modifying genes such as ERAP1/2) constitutes the first hit, an environmental trigger (viral infection or vaccination) provides the second hit through molecular mimicry or bystander activation, and the third hit is the breakdown of ocular immune privilege that permits the inflammatory cascade to proceed (47–49, 62). While mechanistically plausible and consistent with available epidemiological and immunogenetic data, direct causal evidence for the sequential three-hit model remains limited, and it should be regarded as a framework for hypothesis generation rather than an established mechanism. The model is inherently multifactorial and does not privilege any single anatomical site as the primary target; rather, it suggests that the interaction between genetic susceptibility, environmental triggers, and immune privilege may determine both whether disease develops and which anatomical structures are preferentially affected.

## 7 Toward a new classification paradigm

### 7.1 The limitations of morphological classification

The traditional WDS classification, based on the ophthalmoscopic appearance of fundus lesions (white dots, placoid lesions, birdshot lesions, etc.), has proven increasingly inadequate in the era of multimodal imaging (6, 17). Conditions that appear similar clinically have been shown to have fundamentally different pathophysiological bases, while conditions that appear different may share common mechanisms. This fundamental disconnect between morphological appearance and underlying pathology has motivated calls for a paradigm shift in WDS classification.

### 7.2 The MUV task force framework

The Multimodal Imaging in Uveitis (MUV) Task Force has published a series of landmark reports that collectively propose a radical reclassification of the WDS. Fabiani and colleagues, in Report 1, posed the fundamental question of whether it is time to adopt a new nomenclature and classification using multimodal imaging techniques, concluding that the evidence strongly supports such a change (15). This report laid the groundwork for subsequent disease-specific imaging guideline reports.

Report 5 (Gangaputra and colleagues) established evidence- and consensus-based imaging guidelines for multifocal choroiditis with panuveitis (MFC) and punctate inner choroidopathy (PIC) (51). Report 6 (Munk and colleagues) provided analogous guidelines for MEWDS, defining the characteristic imaging signatures across all modalities (38). Report 8 (Pichi and colleagues) addressed birdshot chorioretinopathy, integrating the HLA-A29 molecular data with multimodal imaging findings (68).

The culminating Report 11 (16) explicitly deconstructs the WDS concept and proposes that each non-infectious posterior uveitis (NIPU) entity has distinct anatomic features on multimodal imaging and should no longer be grouped under a single classification (16). Based on multimodal imaging analysis using structural OCT, OCT-A, and ICGA, Report 11 demonstrated that each of the six classical WDS entities exhibits a unique pattern of anatomic involvement:

- **MEWDS**: Primary involvement of the photoreceptor/RPE layer, with hyperreflective lesions extending from the RPE through the EZ, without evidence of primary choroidal vascular occlusion.
- **MFCPU and PIC**: Characterized by breaches in Bruch's membrane with inflammatory communication between the choroid and retina.
- **APMPPE and serpiginous choroiditis**: Primary inflammatory choriocapillaris occlusion, with severe choroidal ischemia demonstrable on ICGA.
- **Birdshot chorioretinopathy**: Diffuse choroidal stromal infiltration originating in the deep choroid, with secondary outer retinal damage, associated with HLA-A29.

These anatomical distinctions were shown to reflect different presumed immunopathogenic pathways, supporting the conclusion that these entities should be managed as distinct diseases rather than grouped under the umbrella of “white dot syndromes” (16).

#### 7.2.1 Reconciling the MUV classification with the MEWDS choriocapillaritis debate

It is important to be explicit about where the MUV framework converges with—and where it diverges from—the three-hypothesis debate reviewed in Sections 3–5. For APMPPE/serpiginous choroiditis, the MUV assignment of primary choriocapillaris ischemia is broadly consistent with the historical Herbort framework and with Deutman's original fluorescein observations (10, 24); for MFC/PIC, the Bruch's membrane breach model supersedes older unitary concepts; and for birdshot, the deep-choroid stromal infiltration model aligns with the HLA-A29 immunogenetic data (48, 62, 68). The principal disagreement concerns **MEWDS itself**. The MUV Report 11, integrating structural OCT, OCT-A, and ICGA across an international multicenter dataset, classifies MEWDS as a photoreceptor/RPE process without primary choriocapillary occlusion (16), a position also reflected in Report 6's OCT criteria (38). This directly contests the Herbort group's contention—based principally on ICGA HDD topography and the low-velocity-flow critique of OCT-A (8, 9)—that MEWDS is a true primary choriocapillaritis continuous with APMPPE. We do not attempt to adjudicate this dispute here; as Sections 3–5 make clear, both positions rest on imaging inference rather than histopathological proof. Across the broader WDS spectrum, disease-specific assignments for APMPPE/SC, birdshot, and MFC/PIC have substantially converged around the MUV anatomical model, and the three competing hypotheses reviewed in Sections 3–5 should be read as MEWDS-focused rather than WDS-general.

### 7.3 Clinical implications

The clinical management implications of this anatomical reclassification are discussed in Section 8.4.

### 7.4 Summary comparison of hypotheses

The three competing hypotheses and their supporting and contradictory evidence are summarized in Table 1.

**Table 1:** Comparison of the three major immunopathogenic hypotheses for MEWDS.

Feature	Choriocapillaritis hypothesis	Photoreceptoritis hypothesis	RPE dysfunction hypothesis	MUV report 11 assignment
Primary site	Choriocapillaris	Photoreceptors (EZ)	Retinal pigment epithelium	MEWDS: explicitly not primary CC occlusion; assigned to photoreceptor/RPE group (16)
Key supporting evidence	ICGA HDDs > clinical lesions; APMPPE–MEWDS continuity	OCT EZ disruption; en-face OCT correlation; functional imaging	FAF hyperautofluorescence patterns; RPE immunoregulatory role	Consistent with MUV 11 assignment to photoreceptor/RPE group based on EZ disruption
ICGA/FA interpretation	True non-perfusion (late HDD) on ICGA	FA: wreath-like hyperfluorescence reflects photoreceptor damage, not choriocapillaris disease	ICGA: impaired ICG uptake by RPE	Partially consistent; MUV 11 acknowledges RPE involvement but classifies under photoreceptor/RPE rather than primary RPE
OCT-A findings	Apparent “normal” due to low-flow detection limit	Preserved choriocapillaris flow	Not specifically addressed by the hypothesis	Used as key evidence against primary CC non-perfusion in MUV 11 reasoning
Explains spontaneous recovery?	Yes—inflammation resolution → reperfusion	Partial—PR regeneration	Partial—RPE metabolic recovery	Imaging-based consensus, not histopathologically validated
Key limitation	No direct histopathological proof	EZ disruption is non-specific; no direct histopathological evidence	ICG–protein complex biophysics argue against; underdeveloped evidence base	MUV Report 11 avoids adjudicating between sub-retinal layers; assignment based on multimodal consensus
Most consistent with	ICGA, clinical-anatomical discrepancy	OCT, en-face imaging, functional correlation	FAF, RPE immunoregulatory biology	Imaging-based consensus across ICGA, OCT-A, FAF; HDDs reinterpreted as RPE uptake dysfunction; wreath-like FA pattern attributed to photoreceptor/RPE changes

CC, choriocapillaris; EZ, ellipsoid zone; HDD, hypofluorescent dark dot; ICGA, indocyanine green angiography; OCT-A, optical coherence tomography angiography; FAF, fundus autofluorescence; PR, photoreceptor; RPE, retinal pigment epithelium; FA, fluorescein angiography.

## 8 Discussion

### 8.1 Synthesis of the evidence

The white dot syndromes encompass conditions with heterogeneous anatomical origins—ranging from primarily choriocapillaris disorders (APMPPE, serpiginous) to entities with still-debated outer-retina/RPE/choriocapillaris localization (MEWDS) to pan-choroidal inflammatory diseases (birdshot, MFC/PIC)—and this anatomical diversity is fundamental to interpreting the evidence discussed below. The three hypotheses reviewed in Sections 3–5—choriocapillaritis, photoreceptoritis, and RPE dysfunction—each account for a recognizable subset of MEWDS observations, but none, taken alone, accommodates the full multimodal imaging phenotype. ICGA-derived hypofluorescent dark dots that extend beyond clinically visible lesions remain the strongest argument for a primary choriocapillary process; OCT-documented ellipsoid zone disruption, en-face OCT correlations, and the concordance of structural with functional deficits support primary photoreceptor involvement; and fundus autofluorescence patterns together with the immunoregulatory biology of the RPE sustain the case for RPE dysfunction. The disagreement is not, for the most part, about what is seen—the imaging signs are reproducible—but about which anatomical layer should be designated as the primary site of injury.

Two observations complicate any simple adjudication. First, each imaging modality interrogates a different biological process and operates at a different spatial and temporal resolution, so apparent discrepancies between ICGA, OCT-A, en-face OCT, and FAF may reflect measurement biology rather than mutually exclusive pathologies. Second, the outer retina–RPE–choriocapillaris complex is functionally and metabolically coupled; injury at one site can propagate rapidly to adjacent layers, making it difficult, on cross-sectional imaging alone, to distinguish a primary lesion from a secondary response.

Accordingly, this review does not attempt to resolve the MEWDS primary-site debate. Its position is deliberately narrower: to map the evidence supporting each hypothesis, to identify the methodological boundaries of that evidence, and to place the MEWDS debate alongside the comparatively well-differentiated mechanisms of other white dot syndromes. The MUV Task Force Report 11 assignment of MEWDS to the photoreceptor/RPE group is an important consensus position, but—as Report 11 itself acknowledges—it is an imaging-based classification rather than a histopathological verdict.

### 8.2 Comparison with MUV task force report 11

The MUV Task Force Report 11 (16) is the most comprehensive current consensus statement on WDS classification and provides the anatomical framework against which any narrative review must be positioned. Report 11 deconstructs the WDS umbrella and assigns MEWDS to a photoreceptor/RPE group characterized by hyperreflective lesions extending from the RPE through the EZ, without evidence of primary choriocapillary occlusion; APMPPE and serpiginous choroiditis are assigned to a choriocapillaris ischemia group, MFC/PIC to a Bruch's membrane disruption group, and birdshot to a deep choroidal stromal infiltration group (16).

The present review complements, rather than duplicates, Report 11 in three ways. First, it places the three MEWDS hypotheses—including the RPE dysfunction hypothesis, which has received less attention in the MUV reports—in direct side-by-side comparison with the disease-specific mechanisms of APMPPE, birdshot, and MFC/PIC, so that the reader can see where the MEWDS evidence converges with and where it diverges from the MUV assignments. Second, it systematically integrates viral triggers, anti-retinal autoantibodies, and HLA genetic susceptibility into the anatomical classification, whereas Report 11 focuses primarily on multimodal imaging criteria. Third, it explicitly declines to adjudicate the primary site of injury in MEWDS and instead maps the evidentiary boundaries and methodological limitations of each position, including the inference-dependent nature of ICGA, OCT-A, and EZ interpretation. Report 11 remains the authoritative classification document; this review is intended as a mechanistic and evidence-synthesis companion to it.

### 8.3 Limitations

The most important limitation of this review—and of the MEWDS literature as a whole—is that virtually all mechanistic claims rest on imaging inference rather than histopathological proof. ICGA hypofluorescent dark dots are commonly interpreted as choriocapillary non-perfusion, but the same appearance can arise from altered dye kinetics, blockage, or impaired RPE uptake; OCT-A flow maps have a finite flow-detection threshold and cannot reliably distinguish absent flow from very slow flow; and ellipsoid zone disruption on OCT is a non-specific sign of photoreceptor stress that occurs across a wide range of retinopathies. To date, no acute MEWDS eye has been examined by biopsy, and—given the self-limited nature of the disease and the risks of ocular tissue sampling—it is unlikely that such specimens will become available. The mechanistic debate is therefore bounded by the resolution and physics of current imaging, and conclusions drawn from it should be framed accordingly.

Second, the autoantibody evidence cited in Section 6.1 derives principally from AZOOR and non-paraneoplastic autoimmune retinopathy cohorts rather than from prospectively characterized MEWDS series (32, 52, 54). Anti-retinal antibodies may be a consequence of retinal injury and subsequent exposure of sequestered antigens rather than a cause, and extrapolation of these findings to MEWDS should be made cautiously. Third, the proposed three-hit model in Section 6.3—HLA susceptibility, viral trigger, and loss of immune privilege—is mechanistically plausible and consistent with available epidemiological and immunogenetic data, but direct causal evidence for the postulated sequence is limited; it should be treated as a working hypothesis rather than an established mechanism.

Fourth, as a narrative review this work did not employ a systematic search strategy, duplicate screening, risk-of-bias assessment, or formal evidence grading. The selection and weighting of studies reflect the authors' judgment and are subject to the usual biases of that approach. Finally, the literature is unevenly distributed across WDS subtypes: MEWDS and birdshot have accumulated substantial bodies of multimodal imaging and immunogenetic work, whereas MFC and PIC remain comparatively under-studied, and conclusions about those entities are correspondingly less secure.

### 8.4 Clinical implications

The observations synthesized here are consistent with current practice patterns but should not be read as a clinical guideline, and this review provides no new treatment data. For MEWDS, where the primary involvement is at the photoreceptor/RPE level (following the MUV Report 11 assignment) and spontaneous recovery is the rule, aggressive immunosuppression does not appear to be routinely required (6, 38). For APMPPE and serpiginous choroiditis, where primary vascular occlusion can threaten irreversible outer retinal ischemia, early systemic corticosteroid therapy is commonly considered, particularly in eyes with central or bilateral involvement (5, 21, 24). For birdshot chorioretinopathy, the chronic progressive course, strong HLA-A29 association, and IL-17–mediated immune response support early disease-modifying immunomodulatory therapy (47, 48, 68). These statements reflect observations in the published literature rather than recommendations derived from comparative trials; management decisions should continue to be individualized on the basis of disease severity, laterality, structural involvement, and patient factors. Notably, the indications for systemic corticosteroids in MEWDS remain debated; most cases resolve spontaneously without intervention, but steroid therapy may be considered in cases with prolonged visual loss, bilateral involvement, or atypical severe presentations. Furthermore, the recently described epidemic MEWDS-like phenomenon (epiMEWDS)—characterized by clusters of MEWDS-like presentations occurring in temporal and geographic proximity—raises intriguing questions about environmental triggers and population-level susceptibility that warrant further investigation.

It is also important to recognize that overlap syndromes, phenocopies, and infectious mimics can produce a WDS-like appearance. Bianco and colleagues emphasized the need to account for atypical and masquerade presentations when applying the new classification (69); Chen and colleagues documented MFC/PIC lesions with MEWDS-like features (12); and Khochtali and colleagues reported an atypical white dot presentation with choriocapillaris ischemia in the setting of latent tuberculosis, underscoring that infectious etiologies should be excluded before assigning an idiopathic WDS diagnosis (70).

### 8.5 Future directions

Five research priorities follow from this synthesis. First, prospective longitudinal studies with serial multimodal imaging (ICGA, OCT-A, en-face OCT, FAF) combined with functional assessments (microperimetry, multifocal and full-field ERG) are needed to establish the temporal sequence of anatomical involvement with sufficient resolution to inform the primary-site debate (6, 38). Second, standardized autoantibody testing protocols—including agreed antigen panels and normative reference values—are required to make cross-study comparisons possible (32, 52, 54). Third, artificial intelligence–assisted quantitative analysis of multimodal imaging may overcome the subjective, qualitative nature of current image interpretation and identify subtle features that escape visual inspection. Fourth, genome-wide association and whole-exome sequencing in larger, multi-ancestry WDS cohorts are needed to extend the HLA-A29/ERAP model developed in birdshot to other subtypes (62). Fifth, the field should continue the move, catalyzed by MUV Report 11, away from the umbrella term “white dot syndromes” toward disease-specific nomenclature that reflects underlying pathophysiology, with ongoing refinement through integration of imaging, immunological, genetic, and clinical data (16).

## 9 Conclusion

After three decades of investigation, the immunopathogenesis of MEWDS—the white dot syndrome with the most actively debated anatomical origin—remains an active and evolving field. The three-hypothesis debate—choriocapillaritis vs. photoreceptoritis vs. RPE dysfunction—is specific to MEWDS and does not generalize across the white dot syndromes. Within MEWDS, current multimodal imaging evidence supports contributions from all three candidate layers but cannot, on its own, identify a single primary site; the MUV Task Force Report 11 assignment of MEWDS to the photoreceptor/RPE group is an important imaging-based consensus, not a histopathological verdict. Beyond MEWDS, APMPPE, birdshot chorioretinopathy, MFC/PIC, and AZOOR are increasingly understood as mechanistically distinct diseases anchored in choriocapillaris ischemia, HLA-A29–driven choroidal stromal inflammation, Bruch's membrane disruption, and outer retinal autoimmunity, respectively. Viral triggers, anti-retinal autoantibodies, and HLA susceptibility interact across these entities in disease-specific patterns, but direct causal evidence for integrated models remains incomplete. Taken together, the available data argue that the umbrella term “white dot syndromes” should be retired in favor of disease-specific, mechanism-driven nomenclature.
